# Supplementary material for: The PTS Components in Klebsiella pneumoniae Affect Bacterial Capsular Polysaccharide Production and Macrophage Phagocytosis Resistance
Source: Microorganisms. 2021 Feb 8;9(2):335. doi: 10.3390/microorganisms9020335 (PMC7914778; doi:10.3390/microorganisms9020335)
Supplement: Supplementary file 1 [file microorganisms-09-00335-s001.pdf]

microorganisms

Supplementary Materials: The PTS Components in *Klebsiella pneumoniae* Affect Bacterial Capsular Polysaccharide Production and Macrophage Phagocytosis Resistance

Novaria Sari Dewi Panjaitan [1,†] Yu-Tze Horng [1,†], Chih-Ching Chien [2], Hung-Chi Yang [3], Ren-In You [1] and Po-Chi Soo [1,*]

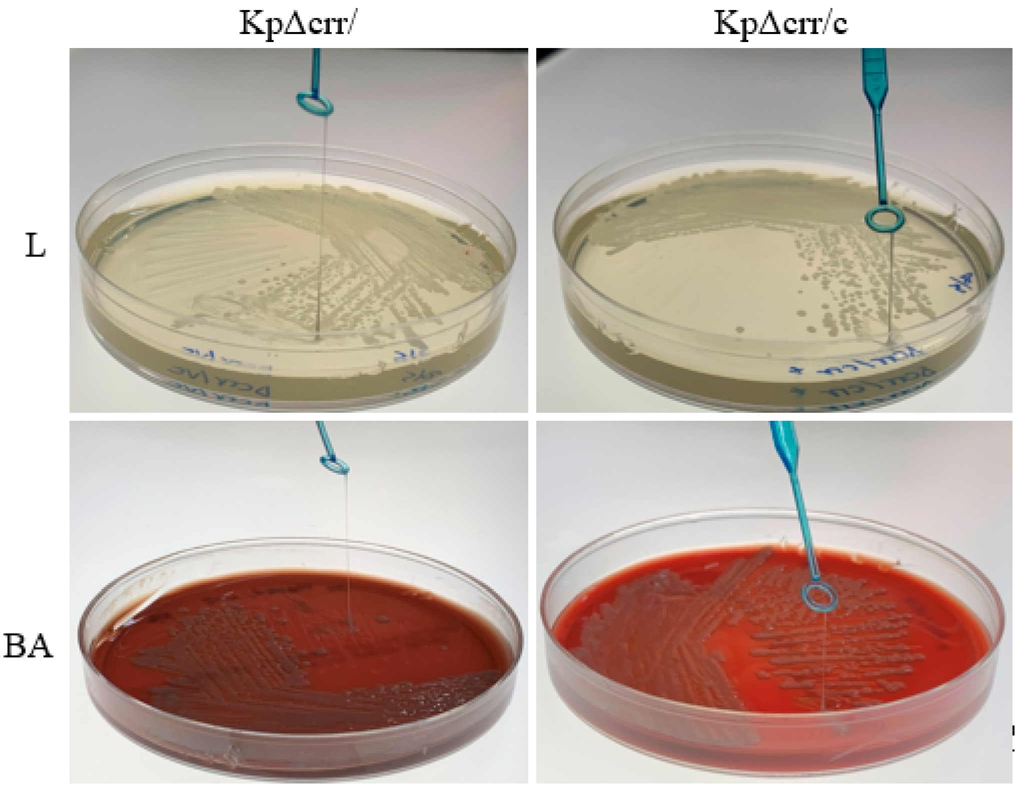

Figure S1. Hypermucoviscosity phenotype of *crr* complemented strain observed by the string test. LB: LB agar plate. BAP: 5% sheep blood agar plate. KpΔcrr/vc: *K. pneumoniae crr* mutant carrying pBAD33 (vector control). KpΔcrr/crr: *K. pneumoniae crr* mutant carrying pBAD33::crr (*crr* complemented strain).

Publisher's Note: MDPI stays neutral with regard to jurisdictional claims in published maps and institutional affiliations.

Copyright: © 2021 by the authors. Submitted for possible open access publication under the terms and conditions of the Creative Commons Attribution (CC BY) license (http://creativecommons.org/licenses/by/4.0/).

Table S1. Bacterial strains and plasmids used in this study.

Strain	Relevant genotype and phenotype	Reference or source
E. coli		
DH5α	F −, ϕ80d*lac*Z1M15 (*lacZYA-argF*) U169, *deoR*, *recA1*, *endA1*, *hsdR17*(rk⁻, mk⁺), *phoA*, *supE44*, λ⁻, thi-1, *gyr*A96, *rel*A1	Invitrogen (Thermo Fisher Scientific, USA)
S17-1 λpir	λ-pir lysogen of S17-1 [*thi pro hsdR⁻ hsdM⁺ recA* RP4 2-Tc::Mu-Km::Tn7 (TpR. SmR.)]. Permissive host able to transfer suicide plasmids requiring the Pir protein by conjugation to recipient cells	a
K. pneumoniae		
STU1	Laboratory-maintained strain, Amp^r	National Taiwan University. b
Δ*crr*	Deletion of *crr* gene in STU1	b
Δ*etcABC*	In frame deletion of *etcABC* genes in STU1	b
Δ*crr*Δ*etcABC*	In frame deletion of *etcABC* genes in Δ*crr*	b
Δ*crr*/*crr*	*crr* complement strain: *K. pneumoniae crr* mutant carrying pBAD33::crr	This work
**Plasmid**		
pBlueScript SK+ (pBSK)	Cloning vector containing *lac* promoter, pUC *ori*, Amp^r	Stratagene (Stratagene California, USA)
pBSK-Gm	pBSK derivative carrying gentamicin resistance gene at the *ScaI* site, Gm^r	c
pBSK-Gm::Km	pBSK-Gm derivative carrying kanamycin resistance gene at the *Sal*I site, Gm^r, Km^r	This work
pBSK-Gm::Km::etcABC	pBSK-Gm::Km derivative carrying *etcABC* genes at the *Sca*I site, Gm^r, Km^r	This work
pBAD33	Expression vector utilizing P$_{BAD}$ promoter, pACYC184 *ori*, Cm^r	d
pBAD33::crr	pBAD33 carrying *crr* at the *Xba*I and *Hind*III sites with poly-His tag (6x His) fused to N-terminus of Crr.	This work
pMV261::ZsGreen	pMV261 carrying fluorescent reporter gene, *ZsGreen*, Amp^r	kind gift from assistant Prof. Yih-Yuan Chen
pBSK-Km	pBSK derivative carrying kanamycin resistance gene at the *SalI* site, Km^r	This work
pBSK-Km::ZsGreen	pBSK-Km carrying *ZsGreen* gene at the *SalI* and *BamHI* sites. *ZsGreen* gene was cloned from pMV261::ZsGreen, Km^r.	This work
pW18mobsacB	Conjugative vector, R6K *ori*, RP4 *mob*, *sacB*, Km^r, suicide plasmid in *K. pneumoniae*.	b

Note:

a R. Simon, U. Priefer., A. Pühler. Bio/Technology 1 784–791, 1983, https://doi.org/10.1038/nbt1183-784.

b N. S. D. Panjaitan, Y. T. Horng, S. W. Cheng, W. T. Chung, P. C. Soo, Front Microbiol 10: 1558, 2019, https://doi: 10.3389/fmicb.2019.01558. eCollection 2019

c Y. T. Horng, C. J. Wang, W. T. Chung, H. J. Chao, Y. Y. Chen, P. C. Soo, J Microbiol Immunol Infect 51: 174-183, 2018, https://doi: 10.1016/j.jmii.2017.01.007. Epub 2017 Jun 29.

d L. M. Guzman, D. Belin, M. J. Carson, J. Beckwith, 177 4121–4130, 1995, https://doi: 10.1128/jb.177.14.4121-4130.1995.

Table S2. Oligonucleotide primers used in this study.

Primer	Sequences (5′ → 3′)	Target / purpose
16S rRNA qPCR FP	GCACAGAGAGCTTGC	*16S rRNA*
16S rRNA qPCR RP	CACTTTGGTCTTGCGA	*16S rRNA*
16S rRNA Probe	/56-FAM/ATGTCTGGG/ZEN/ AAACTGCCTGATGGA/3IABkFQ/	*16S rRNA*

recA qPCR FP	CCGCTTTCTCAATCAGCTTC	*recA* mRNA / RT-qPCR
recA qPCR RP	TTAAACAGGCCGAATTCCAG	*recA* mRNA / RT-qPCR
recA Probe	/56-FAM/TCGCCGTAG/ZEN/ AAGTTGATGCCTTCG/3IABkFQ/	*recA* mRNA / RT-qPCR
etcA qPCR FP	CGCCGAATATGTCAACGAGA	*etcA* mRNA / RT-qPCR
etcA qPCR RP	ATATGACTGTCAGCATCGGC	*etcA* mRNA / RT-qPCR
etcA Probe	/56-FAM/TTATCACCA/ZEN/ CAGCCACGCCAATCT/3IABkFQ/	*etcA* mRNA / RT-qPCR
etcB qPCR FP	GTAACGGTATTGGCAGCTCA	*etcB* mRNA / RT-qPCR
etcB qPCR RP	CAGCAATTTTCATCAGCGCA	*etcB* mRNA / RT-qPCR
etcB Probe	/56-FAM/ACAGTGGGC/ZEN/ CGGAAATTGATTTGC/3IABkFQ/	*etcB* mRNA / RT-qPCR
etcC qPCR FP	ACCGACCAAAACATGCTGAT	*etcC* mRNA / RT-qPCR
etcC qPCR RP	GAGCTGATGAATCCCACCAC	*etcC* mRNA / RT-qPCR
etcC Probe	/56-FAM/TGTTCTTCA/ZEN/ CCTTTAGTCCCACCGC/3IABkFQ/	*etcC* mRNA / RT-qPCR
galF qPCR FP	CAAAGGCAATTCCAAAGGAG	*galF* mRNA / RT-qPCR
galF qPCR RP	CCAGCTCGTAGGAGGTATCG	*galF* mRNA / RT-qPCR
galF Probe	/56- FAM/CGAGGAGTG/ZEN/CGTCACCAGAACAAT/3I ABkFQ/	*galF* mRNA / RT-qPCR
wzi qPCR FP	TGACGGCCAGAACTACC	*wzi* mRNA / RT-qPCR
wzi qPCR RP	GACAACGACTTGCGTAACGA	*wzi* mRNA / RT-qPCR
gnd qPCR FP	TTCACCATCTTCAGCACGAG	*gnd* mRNA / RT-qPCR
gnd qPCR RP	ATGGTGGCAACACCTTCTTC	*gnd* mRNA / RT-qPCR
crr FP	CCCGGGGACGAGTTAATGACGCTGGTT	Amplification of *crr* for Crr complementation
crr RP	AAGCTTGTGCTATCGACCGGTAACCT	Amplification of *crr* for Crr complementation
crr up FP	GGCGGCCGCCCCGGGAAATTG AAGCGCTGCGTAGT	Construction of *crr* deletion used suicide plasmid
crr up RP	GGGGATCCGCATCTCGTGGATTAGCAGA	Construction of *crr* deletion used suicide plasmid
crr down FP	GGGGATCCCGGTAATCCGCATCAAGAAG	Construction of *crr* deletion used suicide plasmid
crr down RP	GGGAGCTCCCCGGGACGCCTATCGTCAGCACCT	Construction of *crr* deletion used suicide plasmid
KO crr 1HR FP	TTGCGATGGATCGTAAAGAG	PCR amplification for confirmation *crr* deletion
KO crr 1HR RP	GGTCACCACCGGCTATATG	PCR amplification for confirmation *crr* deletion
